# Irritable bowel syndrome with diarrhea: time to change practice?

**DOI:** 10.1097/MOG.0000000000001157

**Published:** 2026-02-09

**Authors:** Cristina Caranfil, Luisa Bertin, Fabiana Zingone

**Affiliations:** a Department of Surgery, Oncology and Gastroenterology, University of Padua; b Unit of Gastroenterology, Azienda Ospedale Università Padova, Padua, Italy

**Keywords:** cognitive behavioral therapy, disorders of Gut-Brain interaction, diets, hypnotherapy, neuromodulators

## Abstract

**Purpose of review:**

Traditional approaches to irritable bowel syndrome with diarrhea (IBS-D) relied on extensive exclusionary testing and empiric symptom management. Recent advances in understanding neuroimmune pathophysiology, refined diagnostic algorithms, emergence of novel biomarkers, and clarification of comparative treatment efficacy through systematic reviews necessitate evaluation of whether accumulated evidence warrants substantive changes to contemporary diagnostic and therapeutic practice in IBS-D management.

**Recent findings:**

Diagnostic paradigms have shifted toward symptom-based approaches utilizing judicious testing informed by alarm features, with emerging biomarkers including neutrophil-to-albumin ratio, microRNA-148, and bile acid malabsorption markers showing promise. Therapeutically, tricyclic antidepressants demonstrate robust efficacy as neuromodulators, while selective serotonin reuptake inhibitors show limited benefit. Emerging neuroimmune therapies targeting mast cell activation, including histamine receptor antagonists, represent promising avenues. Low FODMAP and Mediterranean diets demonstrate substantial efficacy, while brain–gut behavioral therapies achieve clinically meaningful improvements in refractory populations through accessible delivery modalities.

**Summary:**

Contemporary evidence supports fundamental practice shifts from exclusionary testing toward targeted investigation of treatable mimics and from empiric management toward mechanism-based multimodal interventions integrating neuromodulators, dietary modifications, and behavioral therapies. Optimal outcomes require individualized treatment selection informed by symptom phenotype and comorbidity profiles, ideally delivered through integrated care models combining gastroenterology, dietetic, and behavioral expertise.

## INTRODUCTION

Irritable bowel syndrome with diarrhea (IBS-D) represents a prevalent disorder of gut-brain interaction affecting approximately 4% of the global population, characterized by recurrent abdominal pain associated with altered bowel habits and loose or watery stools in the absence of organic disease [1^▪^,2^▪^]. The substantial impact on quality of life, work productivity, and healthcare utilization underscores the clinical imperative for accurate diagnosis and effective management, yet both aspects have evolved substantially in recent years [3–5^▪^]. Traditional approaches positioned IBS-D as a diagnosis of exclusion requiring extensive investigation to rule out organic disease, while therapeutic strategies relied predominantly on empiric symptom-directed interventions with limited mechanistic rationale. The past decade has witnessed fundamental transformation across both diagnostic and therapeutic paradigms, with converging evidence revealing complex neuroimmune dysfunction, postinfectious mechanisms, and altered gut–brain axis signaling as central disease drivers [6^▪^]. Historically, IBS-D was often characterized primarily through what it was not rather than what it was, contributing to both diagnostic nihilism and therapeutic uncertainty [7^▪^]. Diagnostic frameworks have shifted toward more judicious testing strategies informed by alarm features and biomarker panels, with growing recognition that conditions such as bile acid malabsorption, microscopic colitis, and exocrine pancreatic insufficiency represent treatable mimics requiring targeted exclusion. Concurrently, systematic reviews and network meta-analyses have clarified the comparative efficacy of established treatments, and the expanding therapeutic armamentarium now encompasses mechanism-targeted agents, microbiome-directed therapies, refined dietary approaches, and integrated behavioral interventions, each with distinct evidence profiles. These advances raise the fundamental question of whether accumulated evidence warrants substantive changes to contemporary clinical practice in IBS-D diagnosis and management. 

**Box 1:**
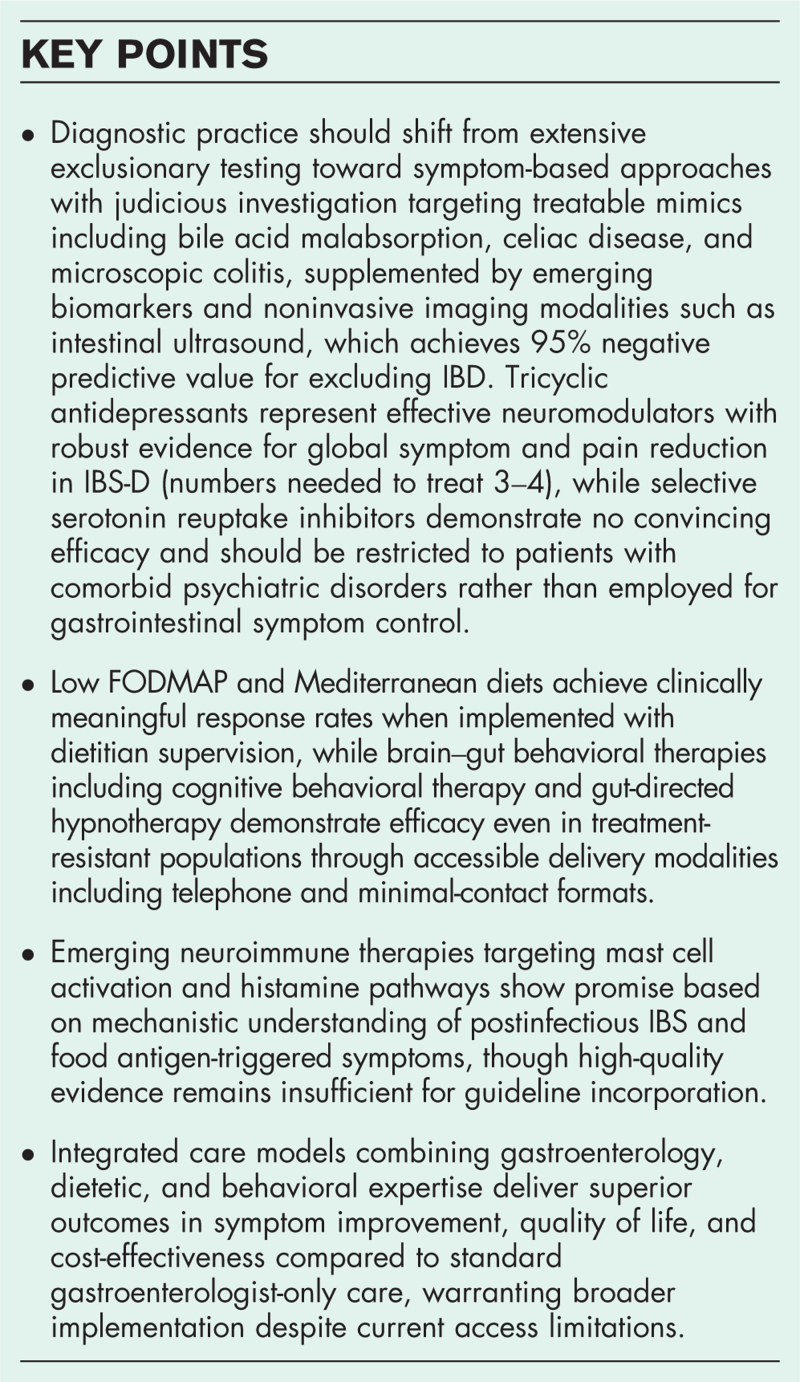
no caption available

## DIFFERENTIAL DIAGNOSIS OF IRRITABLE BOWEL SYNDROME WITH DIARRHEA: EMERGING CONSIDERATIONS

**FIGURE 1:**
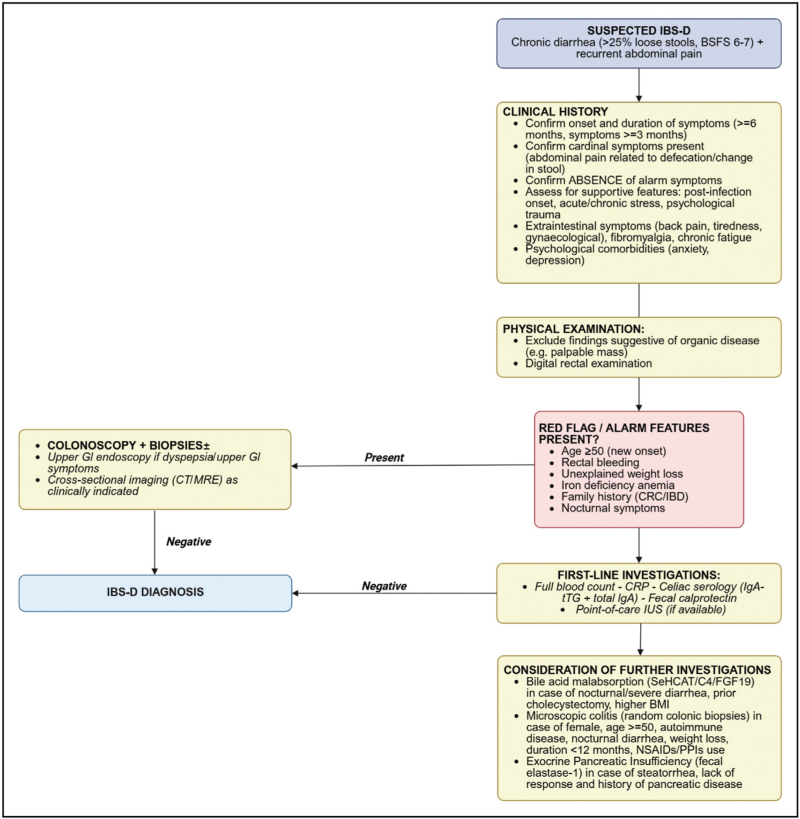
Proposed diagnostic algorithm for the diagnosis of irritable bowel syndrome with diarrhea. BSFS, Bristol Stool Form Scale; C4, 7α-hydroxy-4-cholesten-3-one (serum bile acid precursor); CRC, colorectal cancer; CRP, C-reactive protein; CT, computed tomography; FGF19, fibroblast growth factor 19; GI, gastrointestinal; IBD, inflammatory bowel disease; IBS-D, irritable bowel syndrome with diarrhea; IgA, immunoglobulin A; IgA-tTG, immunoglobulin A tissue transglutaminase (celiac serology); IUS, intestinal ultrasound; MRE, magnetic resonance enterography; NSAIDs, nonsteroidal anti-inflammatory drugs; PPIs, proton pump inhibitors; SeHCAT, 23-seleno-25-homotaurocholic acid (bile acid malabsorption test).

The diagnostic approach to IBS-D has evolved substantially toward symptom-based strategies rather than exclusionary testing. Current IBS management guidelines advocate the use of symptom-based criteria to make a diagnosis of IBS, in combination with limited investigation [8]. The Rome IV, with the next edition scheduled for release in 2026, criteria remain the current gold standard for diagnosis, requiring recurrent abdominal pain on at least 1 day per week over the last 3 months, associated with two or more of defecation-related symptoms, changes in stool frequency, or changes in stool form. Figure 1 represents a proposed algorithm for the diagnosis of IBS-D. In the absence of alarm symptoms, baseline investigations should include a complete blood count, C-reactive protein (CRP), fecal calprotectin, and celiac disease (CeD) serology, while colonoscopy should be reserved for patients presenting with alarm features such as anemia, hematochezia, or weight loss [8,9^▪^]. Despite these advances in diagnostic algorithms, specific biomarkers for IBS remain elusive, and the condition can often overlap clinically with other gastrointestinal disorders such as CeD or inflammatory bowel disease (IBD). Importantly, in a 4-year follow-up study of these patients, the miss rate for future organic gastrointestinal disease among those re-referred and re-investigated was only 1% [9^▪^].

CeD warrants exclusion given its symptomatic overlap with IBS and the preventable long-term complications associated with untreated disease through implementation of a gluten-free diet (GFD) [10]. A notable development in recent guidelines permits CeD diagnosis based on positive serology alone (nonbiopsy approach) in specific patient cohorts, which is anticipated to streamline the diagnostic workup for gastrointestinal disorders presenting with diarrhea and reduce the burden of endoscopic procedures [11^▪▪^]. A recently published systematic review and meta-analysis demonstrated that patients with IBS have a significantly elevated risk of celiac seropositivity compared with healthy controls [odds ratio (OR) 4.42; 95% confidence interval (CI), 2.82–6.92], with no significant differences observed between genders or IBS subtypes [12^▪^]. The aggregated seroprevalence of CeD among IBS patients was estimated at 6% (95% CI, 5–8%), with a comparatively higher, though not statistically significant, prevalence noted in IBS-D (OR 1.78; 95% CI, 0.99–3.20) [12^▪^]. Regarding IBD, fecal calprotectin has an established role in its differentiation from IBS. A threshold of 50 μg/g has been shown to differentiate IBD from brain–gut disorders with a pooled sensitivity of 85.8% (95% CI, 78.3–91%) and specificity of 91.7% (95% CI, 84.5–95.7%), achieving a negative predictive value of 99.8% at a 1% IBD prevalence [13^▪^]. However, fecal calprotectin can also be elevated in older or obese patients, in infections or malignancy, or by drugs, including nonsteroidal anti-inflammatory drugs or proton pump inhibitors [9^▪^]. When exceeding 250 μg/g and combined with the Red Flags Index, fecal calprotectin achieves a sensitivity of 100% (29–100%), specificity of 72% (55–85%), positive predictive value of 21% (5–51%), and negative predictive value of 100% (88–100%) in patients with suspected Crohn's disease [14]. It should be noted that fecal calprotectin may poorly correlate with active ileal inflammation and is less sensitive for small bowel disease, whereas C-reactive protein is more specific but less sensitive for detecting intestinal inflammation [13^▪^].

Bile acid malabsorption (BAM) represents another important condition requiring exclusion. The diagnosis should be considered in any patient with suspected IBS-D who reports nocturnal or severe diarrhea, or in anyone who has had a prior cholecystectomy [9^▪^]. Several potential alternative biomarkers to 75-selenium homocholic acid taurine (SeHCAT) for BAM diagnosis exist, including 7α-hydroxy-4-cholesten-3-one (C4) and serum fibroblast growth factor 19 (FGF19).

Noninvasive imaging modalities are receiving increased attention in IBS diagnosis. A monocentric, self-controlled, prospective study demonstrated that panenteric capsule endoscopy in patients under 50 years with suspected IBS (Rome IV criteria) achieved high negative predictive value for significant gastrointestinal diseases and was well tolerated by patients [15^▪^].

Intestinal ultrasound (IUS) has emerged as a valuable point-of-care tool for differentiating brain–gut disorders from IBD: when used as a first-line test with a high negative predictive value of 95%, meaning a negative IUS result makes IBD unlikely. The use of IUS as a point-of-care test can optimize and speed up clinical decision-making, with studies showing it detected inflammation or complications in 54.3% of asymptomatic IBD patients, underscoring its utility in excluding active inflammatory disease in patients presenting with IBS-like symptoms. Combined with biomarkers such as fecal calprotectin and CRP, IUS can help guide the diagnostic workup: negative IUS with normal CRP and fecal calprotectin below 50 μg/g suggests IBS or microscopic colitis rather than IBD, potentially avoiding unnecessary colonoscopy [13^▪^].

Furthermore, IUS has shown utility in distinguishing IBS subtypes through fecal loading assessment, with IBS-D patients exhibiting significantly lower fecal loading scores compared to those with IBS with constipation (IBS-C) [16^▪^]. Although routine colonoscopy has low yield in IBS, certain clinical features should prompt consideration of microscopic colitis [9^▪^]. Factors that are associated with microscopic colitis on random colonic biopsy among patients with suspected IBS-D or functional diarrhea included age over 50 years, coexistent autoimmune disease, nocturnal diarrhea, weight loss, a duration of diarrhea less than 12 months, or recent introduction of a new drug such as PPIs or statins.

Regarding biomarkers for IBS diagnosis, several lines of investigation are currently underway. The neutrophil-to-albumin ratio (NAR) has been shown to serve as a reliable biomarker for inflammatory diseases, with Huang *et al.* demonstrating significantly higher values in IBS-D patients compared to healthy controls (1.48 ± 0.3 vs. 1.32 ± 0.17; *P* < 0.01) [17]. Additionally, emerging research has focused on the role of microRNAs (miRNAs) in IBS pathophysiology. Among these, miR-148 has garnered particular attention for its involvement in regulating intestinal barrier function, as it appears to disrupt tight junction integrity within the intestinal epithelium, thereby increasing permeability to water and electrolytes. At a cut-off value greater than 2080, miR-148 demonstrates a sensitivity of 57.69% and specificity of 88.33% for differentiating IBS-D [18^▪^].

Finally, recent investigations have explored the relationship between IBS-D and alterations in brain structure, with potential implications for targeted therapeutic interventions [19^▪^]. Zhang *et al.*[20^▪^] demonstrated aberrant activity within the limbic system in IBS patients, a region implicated in enhanced emotional responsiveness, disrupted viscerosensory processing, and heightened pain recollection. Subsequent functional MRI (fMRI) studies assessing functional connectivity strength (FCS) revealed distinct neural connectivity patterns in IBS patients, characterized by significantly elevated FCS within the left medial orbitofrontal cortex and diminished FCS within the bilateral cingulate cortex/precuneus and middle cingulate cortex [21^▪^]. These neural signatures enabled differentiation between IBS patients and healthy controls with 91.9% accuracy.

## THERAPEUTIC MANAGEMENT OF IRRITABLE BOWEL SYNDROME WITH DIARRHEA: WHAT HAS CHANGED?

The therapeutic paradigm for IBS-D has evolved from empiric symptom management toward mechanism-targeted interventions addressing the complex neuroimmune and gut–brain axis dysfunction underlying this disorder [22^▪^]. Contemporary evidence supports multimodal strategies integrating peripherally acting agents, central neuromodulators, microbiome-directed therapies, behavioral interventions, and dietary modifications, with recent insights into postinfectious mechanisms and local immune activation fundamentally reshaping treatment rationale [9^▪^].

## PHARMACOLOGICAL THERAPIES

Peripherally acting agents constitute first-line pharmacological therapy for moderate to severe IBS-D [8,23^▪^]. Alosetron, a highly selective 5-HT3 receptor antagonist, modulates visceral sensation and intestinal transit through inhibition of extrinsic pain pathways, though restricted availability limits widespread use. In a recently published real-world study, also ondansetron at 8 mg/day, prescribed as an off-label drug, improved fecal consistency and reduced the frequency of bowel movements [24^▪^]. Eluxadoline, a mixed mu-opioid receptor agonist and delta-opioid receptor antagonist, demonstrates FDA approval based on preferential effects on bowel habit normalization over pain reduction [9^▪^,25^▪^]. Bile acid sequestrants including cholestyramine address the approximately 25–30% of IBS-D patients with bile acid malabsorption, a condition characterized by excessive colonic bile acid delivery triggering secretory diarrhea and low-grade mucosal inflammation [9^▪^]. Loperamide, while reducing stool frequency, demonstrates poor efficacy for improving quality of life or overall well being, highlighting the disconnect between isolated symptom reduction and patient-reported outcomes [9^▪^].

The role of centrally acting neuromodulators in IBS-D management reflects growing recognition of the bidirectional gut–brain axis in symptom pathogenesis. The most recent systematic review and meta-analysis of 28 randomized controlled trials involving 2475 patients demonstrates that tricyclic antidepressants significantly reduce global IBS symptoms with a relative risk (RR) of 0.70 and abdominal pain with RR of 0.72, translating to numbers needed to treat of approximately 3–4 [26^▪▪^]. The ATLANTIS trial definitively established low-dose amitriptyline titration as effective second-line therapy, demonstrating significant superiority over placebo with therapeutic gains of approximately 10–15% [27]. These agents exert multifaceted effects including anticholinergic-mediated intestinal transit slowing, modulation of visceral hypersensitivity through descending inhibitory pathways, and improvement of comorbid sleep disturbance affecting up to 60% of IBS patients [25^▪^]. Nocebo effects represent an important clinical consideration when prescribing tricyclic antidepressants, as patients frequently misattribute preexisting symptoms to medication side effects, with symptom severity correlating with anxiety levels rather than drug blood levels or adherence [25^▪^]. Starting at low doses (10–25 mg) with supportive contact during the first 1–2 weeks helps patients overcome these anticipatory effects, avoiding premature discontinuation that may preclude access to effective therapy, particularly important given the heightened visceral sensitivity and anxiety comorbidity characteristic of IBS-D populations [25^▪^].

Selective serotonin reuptake inhibitors (SSRIs) demonstrate inconsistent efficacy in IBS-D, reflecting fundamental pharmacological limitations. While earlier meta-analyses suggested modest benefit with RR of 0.74 for global symptoms, the 2025 systematic review found no convincing evidence of efficacy [26^▪▪^]. This discordance relates to trial heterogeneity and the inherent SSRI profile: selective serotonin transporter blockade without noradrenergic activity confers advantage for anxiety disorders but limited benefit for chronic pain requiring descending noradrenergic pathways. Mechanistic studies reveal SSRIs enhance gastric accommodation and accelerate intestinal transit through augmented serotonergic neurotransmission, increasing colonic contractility while exerting minimal effects on visceral sensitivity thresholds [28^▪^]. This dissociation between prokinetic effects and absent analgesia fundamentally limits SSRI utility in IBS-D, where transit acceleration may exacerbate diarrhea without addressing abdominal pain. Contemporary evidence supports restricting SSRI use to IBS patients with comorbid psychiatric disorders rather than employing these agents for gastrointestinal symptom control [29^▪^]. Serotonin-norepinephrine reuptake inhibitors show promise for pain reduction with improvements in quality-of-life measures, though dedicated IBS-D investigation remains limited [25^▪^,28^▪^]. Evidence regarding neuromodulators in patients with IBS is summarized in Table 1.

**Table 1:** Neuromodulators in irritable bowel syndrome

Drug class	Agents	Mechanism	Global IBS RR (95% CI)	Pain RR (95% CI)	Starting dose	Target dose range	Main adverse effects	Adverse event withdrawals RR	Bowel effect	Key clinical points
TCAs	Amitriptyline, Nortriptyline, Desipramine, Imipramine	5-HT and noradrenaline reuptake inhibition plus antagonism of 5-HT2, 5-HT3, H1, muscarinic-1, alpha-1 receptors and sodium channel blockade	0.70 (0.62–0.80), effective 11 trials, 1144 patients	0.69 (0.54–0.87), effective 7 trials, 708 patients	10–25 mg at bedtime	25–75 mg daily (up to 150 mg)	Dry mouth, constipation, drowsiness, weight gain (RR 8.74), orthostatic hypotension	1.67 (1.08–2.57), significantly higher	Slows transit; helps IBS-D, may worsen IBS-C	First-line for IBS pain. Moderate certainty for global symptoms. Tertiary amines better for IBS-D. Response in 3–4 weeks
SSRIs	Citalopram, Escitalopram, Fluoxetine, Paroxetine, Sertraline, Fluvoxamine	Selective 5-HT transporter blockade	0.85 (0.65–1.10), not significant 6 trials, 312 patients	0.74 (0.56–0.99), modest benefit but very low certainty 7 trials, 324 patients	Variable: Citalopram 10 mg, Escitalopram 5 mg, Fluoxetine 10 mg, Paroxetine 10 mg, Sertraline 50 mg	Citalopram 10–40 mg, Escitalopram 5–20 mg, Fluoxetine 10–40 mg, Paroxetine 10–40 mg, Sertraline 50–150 mg	Nausea, diarrhea, headache, sexual dysfunction, insomnia	Not significantly different from placebo	Enhances propulsive motility; helps IBS-C, may worsen diarrhea	Not first-line for pain. Useful when anxiety dominates. Better for IBS-C. Very low certainty evidence
SNRIs	Duloxetine, Venlafaxine, Milnacipran	5-HT and noradrenaline reuptake inhibition	3.00 (0.13–68.84), not significant 2 trials, 100 patients	0.22 (0.08–0.59), effective but very low certainty 2 trials, 94 patients	Duloxetine 20–30 mg, Venlafaxine 75 mg, Milnacipran 50 mg twice daily	Duloxetine 30–90 mg, Venlafaxine 75–225 mg, Milnacipran 50–100 mg twice daily	Nausea (very common initially), dizziness, dry mouth, sweating, palpitations. Venlafaxine increases BP at >225 mg	Not separately reported	Neutral to inhibitory; minimal anticholinergic effects	Alternative to TCAs for pain. No anticholinergic/antihistaminic effects. Duloxetine easier than venlafaxine. Very low certainty
TNAs	Mirtazapine, Mianserin, Trazodone	Alpha-2 antagonism increases noradrenaline/serotonin; antagonism of 5-HT2, 5-HT3, H1, muscarinic-1	0.49 (0.29–0.80), effective in single trial, 67 patients	3.88 (0.46–32.94), not significant single trial	7.5–15 mg at bedtime	15–30 mg daily	Weight gain (RR 6.0), sedation (very common), dry mouth, orthostatic hypotension (7%)	Not separately reported	May help nausea and early satiety	Limited IBS use. Better for functional dyspepsia. Risk of serotonin syndrome with SSRIs/SNRIs. Very low certainty
Azapirones	Tandospirone, Buspirone	Partial 5-HT1A receptor agonist	0.80 (0.65–0.99), effective in single trial, 100 patients	1.50 (0.26–8.79), not significant single trial	Tandospirone 5–10 mg three times daily, Buspirone 5 mg three times daily	Tandospirone 10–20 mg three times daily, Buspirone 10–20 mg three times daily	Dizziness, headache, nervousness, nausea	Not separately reported	May enhance gastric accommodation	Limited evidence. Single Japanese trial with tandospirone. Useful when anxiety prominent. No sedation or dependence. Very low certainty
Alpha-2-delta ligands	Pregabalin, Gabapentin	Bind alpha-2-delta subunit of voltage-gated calcium channels	0.88 (0.59–1.30), not significant 2 trials, 415 patients	No significant effect 2 trials, 415 patients	Pregabalin 75 mg twice daily, Gabapentin 300 mg once daily	Pregabalin 150–300 mg twice daily, Gabapentin 300–900 mg three times daily	Dizziness, somnolence, peripheral edema, weight gain, ataxia, fatigue	4.15 (1.48–11.67) Significantly higher 2 trials, 407 patients	No significant GI effect	NOT recommended for IBS. No benefit shown. High withdrawal rates. May help neuropathic abdominal wall pain only. Very low certainty
Atypical antipsychotics	Quetiapine, Olanzapine, Aripiprazole, Levosulpiride, Sulpiride	D2 antagonism; variable 5-HT2A antagonism, 5-HT1A agonism, H1/alpha-1/alpha-2/muscarinic-1 antagonism	No RCTs in 2025 meta-analysis	No RCTs in 2025 meta-analysis	Quetiapine 25 mg at bedtime	Quetiapine 25–100 mg daily	Sedation (very common), weight gain, hyperlipidemia, hyperglycemia, metabolic syndrome, diabetes risk	Not applicable	May decrease gastric sensitivity; sulpirides have prokinetic effects	Augmentation only for severe refractory IBS. Add to existing TCA/SNRI at low dose. Requires metabolic monitoring. Very low certainty (case series only)

Modified from Khasawneh *et al.* and Hanna-Jirala *et al.* [26^▪▪^,28^▪^].

RCT, randomized controlled trial; SNRI, serotonin–norepinephrine reuptake inhibitor; TCA, tricyclic antidepressant; TNA, tricyclic noradrenergic antidepressant

## EMERGING NEUROIMMUNE AND MICROBIOME-TARGETED THERAPIES

Emerging recognition of neuroimmune dysfunction has transformed understanding of IBS-D pathophysiology and therapeutic development [30^▪^]. Postinfectious IBS, developing in approximately 10–25% of patients following acute gastroenteritis, demonstrates persistent T-lymphocyte and mast cell infiltration with ongoing mediator release driving visceral hypersensitivity. Mast cells, consistently elevated in proximity to colonic nerves in IBS-D patients, release histamine, serotonin, tryptase, and proteases that directly activate and sensitize nociceptive visceral afferents [31^▪^]. Elegant work demonstrates that local IgE antibody production against dietary antigens during enteric infections results in loss of oral tolerance, with subsequent food antigen exposure triggering IgE-dependent mast cell activation and meal-induced abdominal pain. IgE-positive mast cells localize closer to nerve endings in IBS patients, with proximity correlating to symptom severity, suggesting IBS represents part of a spectrum of food-induced disorders mediated by mast cell activation [32^▪^]. However, mast cell activation can be either IgE-dependent or IgE-independent, the latter occurring through activation of MRGPRX2 [33,34]. Decraecker *et al.*[35] focused on the role of MRGPRX2 in IBS, showing that it is upregulated and may contribute to mast cell activation in the absence of IgE. The involvement of mast cells, or even mast cell activation syndrome (MCAS), should therefore be suspected in patients with refractory IBS, and associated comorbidities, such as hypermobile Ehlers–Danlos syndrome (hEDS), should also be investigated [36]. Gao *et al.*[37] recently showed that in patients with IBS-D following a low-FODMAP (fermentable oligosaccharides, disaccharides, monosaccharides, and polyols) diet, there is a reduction in mast cell recruitment and activation, further supporting the central role of these cells in the pathophysiology of IBS.

In the context of IBS, MCAS should be suspected when patients have recurrent, episodic symptoms involving at least one additional organ system (e.g. dermatologic manifestations such as rashes or urticaria) together with one or more of the following minor criteria: elevation of mast cell mediators in blood and/or urine, such as serum tryptase, plasma prostaglandin D_2_, or histamine; clinical improvement with mast cell–directed therapy; and more than 20 mast cells per high-power field in extracutaneous tissue (e.g. luminal gastrointestinal tract or bladder biopsies) [38].

Mast cell stabilizers demonstrate clinical efficacy supporting this mechanistic framework. Ketotifen decreases visceral hypersensitivity to rectal distention and improves symptoms while reducing terminal ileal mast cell counts [9^▪^]. Disodium cromoglycate reduces abdominal pain and improves stool consistency in pilot studies of IBS-D patients, with benefits associated with reduced markers of mast cell activation [9^▪^]. Histamine receptor antagonists represent a particularly promising therapeutic approach, with H1-mediated sensitization of TRPV1 channels mediating visceral hypersensitivity and a phase 2 trial of ebastine demonstrating significant superiority over placebo for composite outcomes (12 versus 4% responders) in 202 nonconstipated IBS patients [39].

These immune-directed therapies represent an emerging therapeutic avenue but are not yet incorporated into existing guidelines due to lack of high-quality evidence [8].

Microbiome-directed therapies address the alterations in the gut microbiome affecting up to 60% of IBS-D patients, with small intestinal bacterial overgrowth (SIBO) correlating inversely with *Bifidobacterium* abundance and directly with pain severity [40^▪^]. In this field, Di Pierro *et al.*[41^▪^] recently demonstrated that the butyrate-producing probiotic strain *Clostridium butyricum* CBM588 reduced diarrhea episodes by more than 80%, decreased evacuation frequency by 60%, and improved Bristol Stool Scale (BSS) scores by 30%. However, even though probiotics are believed to reduce symptoms, a specific strain cannot be suggested, and rifaximin demonstrates efficacy for global IBS-D symptoms through bacterial population modulation, while specific probiotic strains, particularly *Bifidobacterium* species, show benefits in restoring depleted populations [42^▪^]. It has also been shown that rifaximin is more effective than placebo in IBS without constipation (risk ratio for persistent symptoms 0.84, 95% CI, 0.79–0.90) [43]. To date, there are no clear indications regarding which probiotic strains should be used for the treatment of IBS, and substantial heterogeneity among studies prevents the formulation of specific evidence-based recommendations. However, a recently published meta-analysis comparing multistrain probiotics with placebo showed that multistrain formulations significantly reduced the total IBS-SSS score (mean difference − 43.66; 95% CI, −65.89 to −21.44; *P* = 0.0001; *I*^2^ = 99%) [44^▪^].

Fecal microbiota transplantation demonstrates a paradoxical disconnect between successful microbiota engraftment and clinical efficacy in IBS-D, where patients achieving symptom improvement actually showed microbiota less similar to donors than nonresponders, indicating that microbiota normalization alone is insufficient for therapeutic benefit and that recipient baseline characteristics are critical determinants of treatment response [45^▪^].

There is still a debate over the use of breath testing to diagnose SIBO and guide management in IBS [46]. Glucose or lactulose hydrogen breath tests may be used to support a diagnosis of SIBO in symptomatic IBS patients, but diagnostic accuracy is affected by oro-caecal transit time and lack of universally accepted cut-offs [47]. While some studies suggest baseline breath test positivity may predict a greater likelihood of symptomatic response to rifaximin in IBS-D, results are inconsistent, and rifaximin has been shown to improve symptoms irrespective of SIBO status in several cohorts [48]. Routine breath testing to guide dietetic change or antibiotic treatment is not generally supported, and gastroenterologists are advised to carefully interpret results within the wider clinical context and current guidelines frameworks.

## DIETARY INTERVENTIONS

Dietary interventions demonstrate efficacy through reduction of fermentable substrates and luminal distension, though comparative effectiveness varies substantially across different approaches [49^▪^]. A recent network meta-analysis of 28 trials (2338 patients) found that the low FODMAP diet ranked fourth for global symptom improvement (RR 0.51), while a starch-reduced and sucrose-reduced diet ranked first (RR 0.41), though confidence in most comparisons was low or very low [50^▪^,^51^^▪▪^]. However, a modified FODMAP-light approaches restricting only fructans and galacto-oligosaccharides demonstrated comparable 63% Food and Drug Administration (FDA)-defined response rates with reduced implementation complexity and lower patient burden [52^▪^]. Gluten-free or wheat-restricted diets, pursued by approximately 25% of patients reporting wheat-triggered symptoms, demonstrate unclear efficacy with pooled meta-analyses failing to show significant benefit compared to gluten-containing diets, likely because perceived benefit relates to fructan (FODMAP) reduction rather than gluten protein elimination [​​​​​​​53^▪^]. A recent randomized double-blind crossover trial in IBS patients who perceived benefit from gluten-free diet found no significant differences in symptom worsening between wheat (39% of participants), gluten (36%), and sham challenges (29%), suggesting that expectation effects rather than specific dietary components drive symptom generation in many patients [​​​​​​​^54^^▪▪^]. Broader research on noncoeliac gluten sensitivity indicates that among individuals self-reporting gluten or wheat sensitivity, meta-analyses of controlled challenge studies demonstrate gluten-specific symptom responses in only 16–30% of cases, with most symptoms attributable to fermentable carbohydrates or nocebo effects rather than gluten protein itself [53^▪^]. Mediterranean diet trials demonstrate high adherence rates and symptom improvement with additional benefits for psychological profile including reductions in anxiety and depression, warranting adequately powered randomized controlled trials given its nutritional completeness and broader health benefits. A recent randomized controlled trial in IBS patients with comorbid mild to moderate anxiety or depression demonstrated that Mediterranean diet counseling achieved markedly superior gastrointestinal symptom response rates compared to habitual diet controls, with additional benefits for depression, notably without changes in FODMAP intake or microbiome parameters [55]. Another recent 6-week randomized trial of 139 UK patients with IBS found the Mediterranean diet superior to traditional dietary advice, achieving clinical response in 62 versus 42% of patients (*P* = 0.017), establishing it as a viable first-line dietary intervention [56^▪▪^]. Dietitian supervision remains strongly recommended by international guidelines to optimize personalization, prevent nutritional inadequacies, identify patients developing avoidant/restrictive food intake disorder patterns, and achieve superior therapeutic threshold attainment compared with physician-delivered or self-directed dietary advice, though access limitations due to waiting lists, cost, and insurance coverage persist across healthcare systems [57^▪^].

## BEHAVIORAL THERAPIES AND INTEGRATED CARE

Brain–gut behavioral therapies (BGBTs) represent evidence-based interventions targeting the bidirectional communication between the central nervous system and enteric nervous system, with cognitive–behavioral therapy (CBT), disease self-management, dynamic psychotherapy, and gut-directed hypnotherapy demonstrating the most robust efficacy profiles in IBS-D management. A 2025 network meta-analysis specifically examining abdominal pain outcomes across 42 trials with 5220 participants revealed that minimal contact CBT demonstrated particularly robust efficacy with a RR of 0.55 for pain not improving, while face-to-face multicomponent behavioral therapy and gut-directed hypnotherapy showed RRs of 0.72 and 0.77, respectively, though confidence in the evidence was rated as low to very low due to methodological limitations and publication bias [58^▪▪^]. Among trials recruiting patients with refractory IBS symptoms, telephone-delivered disease self-management and contingency management proved superior to routine care, highlighting the potential for accessible delivery modalities in treatment-resistant populations. Gut-directed hypnotherapy normalizes rectal sensitivity through altered central processing of afferent signals, with individual, group, and self-administered formats demonstrating comparable efficacy [9^▪^]. Notably, behavioral interventions improve quality of life even when gastrointestinal symptom severity remains unchanged, highlighting their role in addressing the broader disability associated with IBS-D. Acupuncture represents an emerging complementary therapy for IBS-D, with a recent multicenter randomized controlled trial of 280 patients with diarrhea-predominant IBS finding that 15 sessions of acupuncture over 6 weeks achieved significantly better response rates than sham acupuncture (57.9 versus 41.4%, *P* = 0.008), with benefits sustained through 18 weeks follow-up and no severe adverse events [59^▪▪^].

Physical activity represents an underutilized therapeutic modality with substantial evidence supporting its efficacy in IBS-D management. Exercise may alter gastrointestinal function through decreased splanchnic blood flow, increased motility, enhanced immune function, and mechanical movement of the gut. Notably, intensity matters considerably, as high-performance athletes often report adverse gastrointestinal symptoms following strenuous exercise, suggesting that vigorous or high-intensity physical activity may exacerbate symptoms in susceptible individuals [60^▪^]. Personalized exercise plans starting with low-to-moderate intensity and gradually increasing duration are recommended, with monitoring of patient responses essential to maximize benefits while minimizing risks.

Yoga interventions have demonstrated moderate to large effect sizes for IBS symptom improvement, with a 2025 systematic review of 10 studies encompassing 7441 participants revealing Cohen's *d* values ranging from 0.37 to 3.60 for studies utilizing the IBS Symptom Severity Scale as the primary outcome [61^▪^].

**FIGURE 2:**
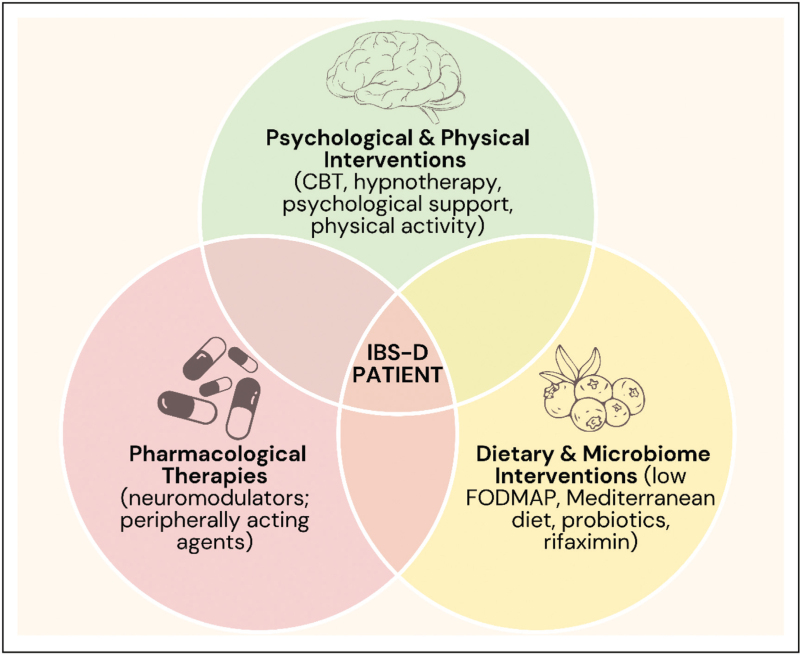
Integrated care in inflammatory bowel disease management. CBT, cognitive behavioral therapy; FODMAP, fermentable oligosaccharides, disaccharides, monosaccharides, and polyols; IBS-D, irritable bowel syndrome with diarrhea.

Contemporary management emphasizes integrated care models wherein multidisciplinary teams combining gastroenterologists, dietitians, and behavioral therapists deliver coordinated treatment. Randomized controlled trials demonstrate that this approach produces superior outcomes in symptom improvement, quality of life, and cost-effectiveness compared to standard gastroenterologist-only care. Figure 2 schematizes this approach.

## CONCLUSION

Contemporary evidence supports fundamental shifts in both diagnostic and therapeutic approaches to IBS-D, moving from extensive exclusionary testing toward judicious investigation informed by alarm features and targeted exclusion of treatable mimics including bile acid malabsorption and microscopic colitis, while therapeutic strategies have evolved from empiric symptom control to mechanism-targeted multimodal interventions integrating central neuromodulators, dietary modifications, behavioral therapies, and emerging neuroimmune approaches. Optimizing outcomes requires individualized treatment selection informed by symptom phenotype and comorbidity profiles, ideally delivered through integrated care models combining gastroenterology, dietetic, and behavioral expertise. However, substantial gaps remain in IBS research. The lack of validated biomarkers forces diagnosis to rely mainly on clinical presentation, thereby limiting patient stratification and targeted treatment and perpetuating a symptom-based management approach. Future research should prioritize improved phenotyping that integrates clinical, microbiome, neuroimmune, and psychosocial factors, along with mechanistic studies of gut–brain and immune pathways. In order to support a shift towards precision medicine in IBS, it is necessary to conduct well designed randomized trials of targeted therapies with long-term outcomes.

## Acknowledgements

*None*.

### Financial support and sponsorship

*None.*

### Conflicts of interest

*F.Z. has served as a speaker for EG Stada Group, Fresenius Kabi, Janssen, Pfizer, Takeda, Unifarco, Malesci, and Kedrion and has served as a consultant for Galapagos. L.B. has served as a speaker for Edra SPA, Takeda. C.C. declare no conflict of interest.*

## REFERENCES AND RECOMMENDED READING

Papers of particular interest, published within the annual period of review, have been highlighted as:

▪ of special interest
▪▪ of outstanding interest
